# Subdiaphragmatic bronchogenic cysts: Case report and literature review

**DOI:** 10.1016/j.ijscr.2025.111235

**Published:** 2025-03-29

**Authors:** Matin Vahedi, Ali Motamedi Rad, Elham Nazar, Alireza Samimiat

**Affiliations:** a Department of General Surgery, Sina Hospital, Tehran University of Medical Sciences, Tehran, Iran; b School of Medicine, Tehran University of Medical Sciences, Tehran, Iran; c Department of Pathology, Sina Hospital, Tehran University of Medical Sciences, Tehran, Iran

**Keywords:** Bronchogenic cyst, Subdiaphragmatic, Subdiaphragmatic bronchogenic cyst

## Abstract

**Introduction and importance:**

Bronchogenic cysts are rare congenital malformations of the respiratory tract, arising from abnormal budding of the foregut during embryogenesis. Clinical manifestations vary by location and complications, ranging from asymptomatic to causing respiratory distress in newborns or recurrent respiratory issues in adults. Subdiaphragmatic bronchogenic cysts are extremely rare, with only a limited number of case reports published. They often present without symptoms or with nonspecific symptoms such as abdominal pain. Diagnosis typically relies on histopathologic analysis of excised biopsies performed during surgery. Due to their rarity and lack of distinctive clinical features, these cysts pose significant diagnostic challenges.

**Case presentation:**

A 36-year-old Iranian man, presented with a 5-month history of abdominal pain. Abdominal sonography revealed a cystic lesion posterior to the liver. An aspiration biopsy indicated an inflammatory process. An abdominal CT scan without contrast reported a right subdiaphragmatic cyst measuring 68 × 52 × 48 mm with a pressure effect on the liver. The diagnosis was uncertain. The cyst was surgically removed, and histopathologic studies confirmed it to be a bronchogenic cyst. The patient had an uneventful recovery with no recurrence after six months.

**Clinical discussion:**

In addition to presenting our case report, we reviewed recent literature and added 24 new cases to the previously identified 100 cases of subdiaphragmatic bronchogenic cysts. Subdiaphragmatic bronchogenic cysts are rare lesions with no specific presentation, making diagnosis extremely challenging.

**Conclusion:**

SBC is a benign lesion. Most patients are asymptomatic; however, due to the favorable prognosis following resection surgery, it remains the optimal management strategy.

## Highlights

• The article focuses on subdiaphragmatic bronchogenic cysts, a rare congenital anomaly of the respiratory system, typically found below the diaphragm.
• A case report of a 36-year-old Iranian man with abdominal pain, diagnosed with a subdiaphragmatic bronchogenic cyst after imaging and surgical intervention.
• Due to their rarity and nonspecific symptoms, these cysts pose significant diagnostic challenges.
• Complete surgical resection is the optimal treatment strategy, with an excellent prognosis post-surgery.
• The article reviews 24 new cases of subdiaphragmatic bronchogenic cysts.

## 1 Background

Bronchogenic cysts are uncommon congenital malformations of the respiratory tract, resulting from anomalous budding of the foregut during embryogenesis [1,2]. Although the exact pathogenesis remains unknown; the hypothesis of embryo abscission and translocation is widely accepted. During the early stages of embryo development, the thorax and abdomen cavity become integrated, and the primary tracheobronchial tree forms by the 5th week. Within this critical period, various abnormal germ cells detach and migrate. Due to the inability to discharge secretions effectively, a bronchogenic cyst develops at the site of migration [3]. They can be found in the mediastinum or the lung parenchyma, depending on the stage of embryological development they originate from. Mediastinal cysts arise earlier in development and peripheral ones (lungs) develop later [4].

The clinical manifestations of affected patients vary according to their location and associated complications. Although they can be asymptomatic [5], and discovered incidentally on radiological imaging, they may become symptomatic in infancy or adulthood. In adults, bronchogenic cysts may cause recurrent coughing, wheezing, or pneumonia [6]. In newborns, they may lead to life-threatening conditions, such as respiratory distress, cyanosis, and feeding difficulty [7,8].

On chest radiography, they present as sharp-defined, solitary, round, or oval-shaped masses with water-density opacities. They may have air-fluid levels, due to previous or current infection. The density of lesions in the CT scan varies from water density to high density because of the blood, calcium, or protein content of the fluid in the cysts. MRI is better than a CT scan at determining the anatomical relations of the cysts, their signal intensity on MRI, also depends on the cyst content [9,10].

In gross pathology, they are Spherical, Smooth, White or pinkish, Single or multiple, ranging from 2 to 12 cm in diameter, frequently unilocular, and filled with clear fluid or proteinaceous mucus, and rarely air or hemorrhagic secretions. Calcification of the cyst wall is uncommon, and communication with the bronchial tree is rare [2]. Histologically, the cysts are lined with columnar respiratory epithelium with occasional areas of squamous metaplasia. The wall may contain airway components such as cartilage plates, bronchial glands, and smooth muscle [2].

Due to the potential risk of complications such as infection, rupture, bleeding, and malignant transformation, complete surgical resection even for asymptomatic patients is recommended [11]. Recurrent cyst aspiration may be an option, but due to the high risk of recurrence, it is not widely accepted. The prognosis, after the cyst resection is excellent [12,13].

Subdiaphragmatic bronchogenic cysts are exceptionally rare, and only a limited number of case reports have been published to date. The clinical presentations of subdiaphragmatic bronchogenic cysts are typically nonspecific, primarily attributed to complications such as local compression and infection. Depending on their location that may cause abdominal pain, dysphagia, chest pain, and dyspnea [14]. Due to the absence of specific radiographic features, they may be easily mistaken for other common diseases of the same origin. These cysts pose challenges in preoperative diagnosis due to their lack of distinctive clinical features, as well as inconclusive findings from laboratory tests and imaging. Their rarity further complicates the diagnostic process, necessitating histopathological examination for definitive diagnosis [15].

In this article, we report another rare case of an ectopic bronchogenic cyst in the subdiaphragmatic space. The patient presented with abdominal pain and the pathology report confirmed a bronchogenic cyst. In addition, we conducted a comprehensive review of recent literature concerning subdiaphragmatic bronchogenic cysts. These cases have been reported after the review conducted by Xiao, J et al (Table 1).

**Table 1:** Demographics, clinical symptoms, imaging features, surgical and follow-up information of literature review and our case.

Case	Year	Age	Sex	Symptoms	Cyst number	Cyst localization	Size (cm)	CT features	Preoperative diagnosis	Surgical procedure	Content of cyst	Follow up
1	2022	76	M	Abdominal pain, distention	1	Lesser gastric curvature	31*31	Well define soft tissue mass	Gastrointestinal stromal tumor	Laparoscopy	Mucoid	N/A
2	2022	39	F	Upper abdominal pain	1	Retroperitoneal space	50*26	Cystic mass	Lymphatic cyst	Laparoscopy	N/A	N/A
3	2022	46	F	Abdominal pain	1	Large curvature	42*41	Uniform mass	Gastrointestinal stromal tumor	Gastrectomy	Turbid fluid	N/A
4	2022	49	F	None	1	Left adrenal gland	54*40	Moderate density cyst	Left adrenal tumor	Laparoscopy	Gelatinous	6 m
5	2022	40	F	Intermittent upper abdominal pain	1	Left inner lobe of liver	32*16	Elliptical low-density cyst	Gallbladder diverticulum	Laparoscopy	Light yellow turbid fluid	6 m
6	2022	60	M	None	1	Left upper abdominal quadrant	150*120*120	Multifocal cystic mass	Lesser momentum malignancy	Laparoscopy	Viscous mucus	1y
7	2022	52	F	None	1	Above pancreas	39*26	N/A	N/A	Laparoscopy	N/A	N/A
8	2022	57	F	None	1	Left adrenal	22*58	Ovoid, well-defined, heterogenous	Benign lesion	Laparoscopy	Mucoid	6 m
9	2022	38	F	Left upper abdominal pain	1	Left suprarenal space	63*25*55	Well-defined, ovoid, heterogenous lesion	N/A	Laparoscopy	Mucoid	N/A
10	2022	35	M	None	1	Lower left of diaphragm	50	Well-defined cyst	Lymphangioma	Laparoscopy	N/A	3 m
11	2023	45	F	Upper abdominal pain	1	Left upper abdominal quadrant	30*20	Cystic mass	Gastrointestinal stromal tumor	Laparoscopy	Gelatinous and mucoid	12 m
12	2023	50	M	N/A	1	Posterior gastric fundus wall	25*30	Lightly low-density nodule	Schwannoma or stromal tumor	Gastroscopic exertion	N/A	N/A
13	2023	37	F	None	1	Cardia of stomach	30	Cystic mass	Gastric duplication, foregut cyst or bc	Laparoscopy	Bright yellow fluid	9 m
14	2023	47	M	None	1	Cardia of stomach	50	Homogeneous low-density lesion	Gastric bc	Laparoscopy	N/A	6 m
15	2023	37	M	None	1	Posterior wall of the stomach,	35	Cystic mass	Bc	Laparoscopy	Yellow-brownish liquid	3 m
16	2023	67	M	None	1	Liver and gastric ligament	31*36	Round, slightly, low-density	Gastrointestinal stromal tumor	Laparoscopy	N/A	6 m
17	2023	65	M	Belching, intermittent epigastric pain	1	Cardia of stomach	40*30	Cystic mass	N/A	Laparoscopy	Coffee colored liquid	2 m
18	2023	41	F	Diastolic arterial hypertension	1	Left adrenal	52*28	Heterogeneous mass	Adrenal mass	Laparoscopy	Serous	N/A
19	2023	54	M	None	2	Left diaphragmatic crura	47*32	Irregular mass shadow	N/A	Laparoscopy	Gelatinous	2 y
20	2023	47	M	Right chest pain	1	Posterior wall of stomach	80*70	Elliptical homogeneous mass	N/A	Laparoscopy	Yellow liquid	2 m
21	2023	40	F	None	1	Left adrenal	55*50*35	Mass and cystic lesion	Adrenal mass	N/A	Gelatinous	N/A
22	2023	65	F	N/A	1	N/A	N/A	N/A	N/A	N/A	N/A	N/A
23	2024	48	F	Frequent urination	1	Right side of pelvic	99*57	Cystic mass	Ovarian cyst, cystic mass	Laparoscopy	Brown sticky fluid	N/A
24	2024	18	M	Recurrent nephrolithiasis, severe left back pain, recurrent abdominal pain	1	Left suprarenal	96*69	Homogenous, thin walled, well circumscribed	Adrenal cyst, gastrointestinal duplication cyst, and adrenal neoplasm	Laparoscopy	White creamy fluid	N/A
Case	2024	36	M	Upper abdominal pain	1	Right sub diaphragm	68*52*48	Well define cyst	Diaphragm mass	Laparotomy	Brown turbid fluid	6 m

This study has been reported in line with the SCARE criteria [40].

## 2 Case presentation

**Fig. 1:**
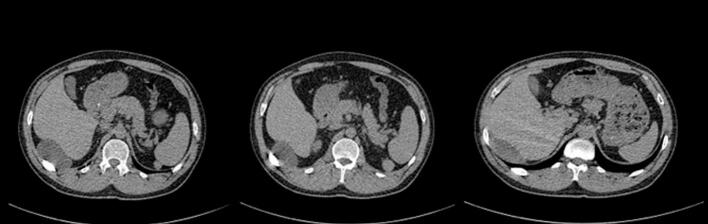
Abdominal CT scan W/O contrast was done which a cystic lesion with the size of 68*52*48 in the right sub diaphragm.

A 36-year-old Iranian man presented with the chief complaint of abdominal pain about 5 months ago preferably the upper part of the abdomen. The nature of the pain was vague and occasional and did not spread anywhere. Hematochezia, melena, anorexia, vomiting, nausea, or fever were not present. He had no significant previous medical history or drug history. He was a smoker (30 Pack/year) and alcoholic. Physical examination revealed 129/700 mmHg blood pressure, a temperature of 36.9 °C, and heart and respiratory rates of 80 and 16 per minute, respectively. Examination of the abdomen showed symmetry without any scars. The abdominal assessment revealed no tenderness and no rebound tenderness, and auscultation detected normal intestinal sounds. The complete blood count (CBC) reported white cell and platelet counts of 5.1 × 10^3^/μL and 246 × 103/μL, respectively. Hemoglobin was 16.5 g/dl at admission. The abdominal sonography was done which reported a cystic lesion with a benign appearance with the size of 75*65*30 in the posterior of the liver and lateral to Perirenal space, about 70 cc brownish liquid aspiration was done which was sent for cytology and analysis. The cystic lesion fluid cytology reported an acute inflammatory process (consisting of abscess material), and no malignant cell was seen. Furthermore, an abdominal CT scan W/O contrast was done and a cystic lesion with the size of 68*52*48 in the right sub diaphragm with pressure effect on the right lobe of the liver was reported (Fig. 1).

**Fig. 2:**
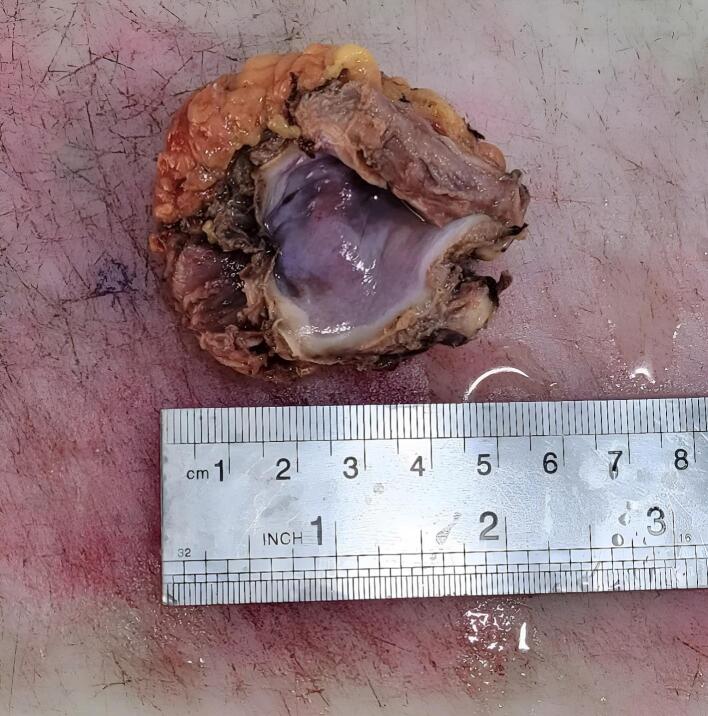
Sub diaphragmatic cystic mass completely surgically removed.

**Fig. 3:**
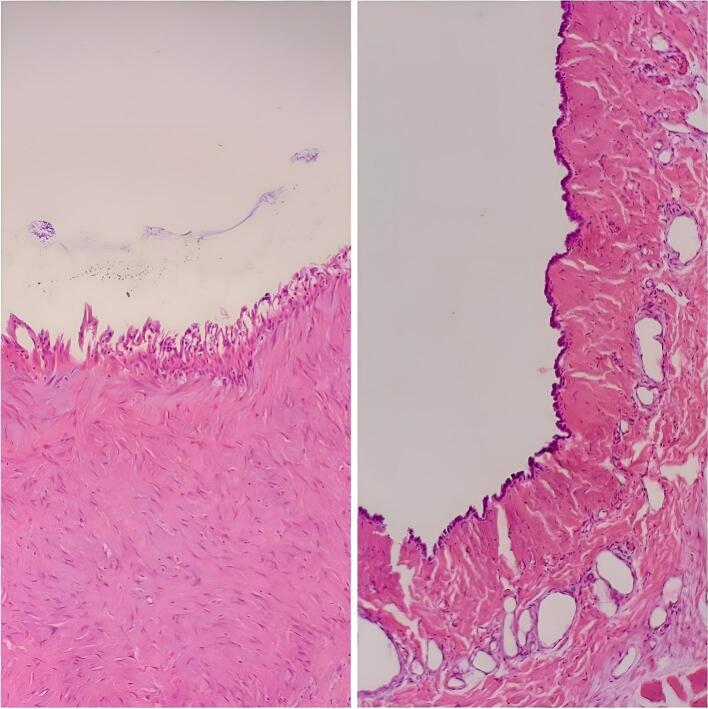
Histopathologic examination showed cystic lesion lined by flat and columnar epithelial cells and variable numbers of goblet cells (H&E X400).

A laparotomy plan was considered for the patient. The mass was completely removed after being freed from the adhesion of the surrounding tissues and it was sent for pathology evaluation. (Fig. 2). Pathological assessment of the extracted tissue pointed to a Benign cystic lesion lined by flat to low columnar epithelial cells and a variable number of goblet cells overlying the congested fibromuscular wall, compatible with bronchogenic cyst (Fig. 3). A three-day hospital discharge followed the patient's uneventful recovery. We performed follow-ups for the patient at the hospital outpatient department at 3-month intervals. The follow-up after 6 months showed that the patient had no signs of recurrence.

## 3 Discussion

Bronchogenic cysts are rare congenital abnormalities that originate during embryogenesis. While the thorax and mediastinum are their typical locations, they occasionally manifest in the subdiaphragmatic space [[1], [2], [3]]. In the most recent review article, approximately 100 cases of subdiaphragmatic bronchogenic cysts were identified in the English literature [15]. This article contributes an additional 24 cases, sourced from 21 articles and letters published between April 2022 and April 2024 [14,[16], [17], [18], [19], [20], [21], [22], [23], [24], [25], [26], [27], [28], [29], [30], [31], [32], [33], [34], [35]]. Furthermore, we present a case from our own institution.

Although rare instances of subdiaphragmatic bronchogenic cysts (sBCs) have been discovered during pregnancy and childhood [7,8,36]. The majority of cases in this review were individuals between 40 and 60 years old, which is consistent with the previous review. Regarding gender distribution, the number of male cases slightly exceeds that of females, mirroring the findings of the last review [15].

Except for one case [32], all other cysts were solitary. The outlier was a 54-year-old male, referenced in a Chinese letter, who simultaneously had a mediastinal bronchogenic cyst (BC) and a subdiaphragmatic bronchogenic cyst (sBC). There was another unusual case in previous *literature where* multiple cysts were present, either unilaterally or bilaterally, on the same side or both sides of the diaphragm.

Cyst sizes varied from 2.0 cm to 15.0 cm, with the majority ranging between 3.0 cm and 5.0 cm, according to the data available in this review. It has been reported that subdiaphragmatic bronchogenic cysts (sBCs) are most commonly found within the size range of 3 cm to 7 cm. The majority of the cysts were located in the left upper abdominal cavity and left retroperitoneal space, in close proximity to the left adrenal gland and pancreas, and around the gastric wall. There was a unique case where an sBC was discovered in the left inner lobe of the liver, a finding that was reported for the first time [20].

A significant number of cases were asymptomatic, while others presented with nonspecific symptoms such as intermittent abdominal pain. Subdiaphragmatic bronchogenic cysts (sBCs) may become symptomatic due to compression at the location or infection. One case exhibited diastolic hypertension, likely due to the cyst's compressive effect on the renal artery [31]. Another case involved an intestinal bronchogenic cyst (BC) that led to frequent urination due to bladder compression [34]. A case reported in the previous review presented with hypertension [37], and another case became symptomatic due to a cyst infection [15].

In most instances, laboratory findings and tumor markers do not contribute significantly to the diagnosis as they are predominantly negative. However, there are rare instances where tumor markers such as CA19–9, CEA, AFP, CA47–7, and CA72–4 have tested positive [21,22,26,28,33,34]. Nonetheless, only one case exhibited malignant changes following surgical biopsy [21].

Imaging studies often do not provide definitive diagnostic information due to their nonspecific findings. Our review, consistent with previous studies, has determined that the density of sBC on CT scans can vary from low to high, with or without enhancement. Additionally, other imaging modalities such as ultrasound (US), endoscopic ultrasound (EUS), and MRI have not proven helpful in diagnosis.

Histopathological findings are crucial for diagnosis. All cysts examined exhibited column-ciliated epithelium. Additionally, cartilage, smooth muscle, and bronchial glands were observed. Only two cases underwent biopsy before surgery [28]. Given that the majority of sBCs are benign, a biopsy could potentially obviate the need for surgery, although those two cases terminated to surgery too. However, it should be noted that biopsies may not always be diagnostic due to issues related to sampling [38]. In the reviewed literature, all cases underwent surgical intervention, with the majority being performed via retroperitoneal laparoscopy.

Despite preoperative studies indicating that sBC is a benign lesion, predicting the likelihood of malignancy remains challenging. Additionally, some cases presented with symptoms, and patients were discharged promptly following surgery. Therefore, surgery continues to be the preferred treatment option.

Following resection, all reviewed cases demonstrated no recurrence. In the most recent review, only one case of malignancy recurred 14 months post-surgery. Thus, patients with sBC generally had a favorable prognosis [39].

## 4 Conclusion

We have added 24 additional cases to the previously reviewed article. Combined with the earlier findings, it is evident that sBC is a benign lesion predominantly occurring in the left adrenal or pancreatic space. Most patients are asymptomatic; however, due to the favorable prognosis following resection surgery, it remains the optimal management strategy.

## Author contribution

MV conceived the study. AS and AM contributed to the case collection, discussion, literature review, and first manuscript draft. MV, AS and AM provided critical revisions. All authors contributed to the article and approved the submitted version.

## Consent for publication

Written informed consent was obtained from the patient to publish this case report and accompanying images. On request, a copy of the written consent is available for review by the Editor-in-Chief of this journal.

## Ethics approval and consent to participate

Ethical review and approval were not required for the study on human participants in accordance with the local legislation and institutional requirements. The ethics committee waived the requirement of written informed consent for participation.

## Guarantor

Alireza Samimiat.

## Research registration number

Not applicable.

## Funding

We do not have any financial support for this study.

## Declaration of competing interest

The authors declare that the research was conducted in the absence of any commercial or financial relationships that could be construed as a potential conflict of interest.

## Data Availability

For data available upon request: “The data that support the findings of this study are available from the corresponding author upon reasonable request.”
